# Associations between anterior segment biometry and high axial myopia in 3438 cataractous eyes in the Chinese population

**DOI:** 10.1186/s12886-022-02300-6

**Published:** 2022-02-12

**Authors:** Ao Miao, Yating Tang, Xiangjia Zhu, Dongjin Qian, Tianyu Zheng, Yi Lu

**Affiliations:** 1 Eye Institute and Department of Ophthalmology, Eye & ENT Hospital, Shanghai Medical College, Fudan University, Shanghai, 200031 China; 2 NHC Key Laboratory of Myopia (Fudan University); Key Laboratory of Myopia, Chinese Academy of Medical Sciences, Shanghai, 200031 China; 3 Shanghai Key Laboratory of Visual Impairment and Restoration, Shanghai, 200031 China

**Keywords:** Anterior eye segment, High myopia, Ocular biometry, Corneal topography, Intraocular pressure

## Abstract

**Background:**

To investigate the associations between anterior segment biometry and high axial myopia in cataractous eyes in the Chinese population.

**Methods:**

Data on 3438 eyes from 3438 subjects were analyzed in this cross-sectional study. Anterior segment biometry, axial length measurements, and intraocular pressure evaluation were implemented using an Oculus Pentacam HR, a Zeiss IOLMaster 500, and a Nidek TonoRef II, respectively. A multivariate-adjusted logistic model and a multivariate-adjusted linear model were used for statistical analysis.

**Results:**

The mean age of the subjects was 62.2 ± 10.6 years, and 56.4% were female. There were 2665 subjects with high axial myopia (axial length, ≥26.50 mm) and 773 without (axial length, < 26.50 mm). The characteristics independently associated with high axial myopia included lower total corneal refractive power, a more negative Q value, greater total corneal astigmatism, greater white-to-white corneal diameter, greater anterior chamber depth, and higher intraocular pressure (all *P* <  0.05). In addition, greater axial length correlated with a thicker temporal cornea and a thinner nasal cornea (both *P* <  0.001).

**Conclusions:**

For cataractous eyes, high axial myopia was associated with corneal flattening, increased total corneal astigmatism, anterior segment enlargement, and intraocular pressure elevation. The findings may inform the choice of intraocular lenses and the calculation of their power, help improve the surgical practice of refractive cataract procedures, and provide useful information on the centration and stability of intraocular lenses.

## Background

The past two decades have witnessed the evolution of cataract surgery into a refractive procedure. For today’s people, cataract surgery is aimed not only to safely remove the opaque crystalline lens, but also to yield postoperative refractive success. However, the postoperative refractive accuracy of cataract surgery in high axial myopia is still challenging [1]. The use of third-generation intraocular (IOL) power formulas, without adjustment, would generally result in unwelcome postoperative hypermetropia in long eyes. Despite the applicability of fifth-generation formulas for eyes of varied ocular dimensions, [2, 3] the refractive outcomes in people with high axial myopia after cataract surgery are still far from perfect.

To yield postoperative refractive success, accurate assessments of the anterior segment are of great importance both in planning of cataract surgery as well as for analyzing postoperative outcomes. Anterior segment biometry refers to the structural and optical characteristics of the anterior eye segment. The parameters measured in anterior segment biometry generally include corneal thickness (CT), corneal refractive power (CRP), corneal astigmatism (CA), white-to-white corneal diameter (WTW), anterior chamber depth (ACD), and corneal asphericity (Q value), among others. Anterior segment biometry plays an important role in all stages of refractive cataract procedures. For IOL power calculation, CRP is a fundamental parameter used in almost all IOL formulas, while other anterior segment biometric parameters are crucial for the optimization of IOL formulas. By using parameters such as ACD, WTW, and CT, fourth- and fifth-generation formulas have generally achieved higher accuracy than formulas from prior generations [2]. Moreover, accurate measurements of CA and ACD are important for calculating the astigmatic power of toric IOLs in patients with high refractive astigmatism. In recent years, the effect of the Q value on IOL power calculation has also attracted increasing attention for the significant link between corneal asphericity and refractive outcomes of IOL implantation [4]. In addition to the implications for IOL power calculation, anterior segment biometric parameters also could give hints for the choice of IOL [5, 6]. A large WTW and a deep ACD are indicative of a large capsular bag with a tendency toward poor IOL stability, [5] in which case a plate-haptic IOL may be suggested to prevent IOL dislocation [6]. Furthermore, anterior segment biometric parameters are helpful to surgical planning. The axes of CA could also inform the selection of the main incision for cataract surgery, as a main incision in the steep meridian may be helpful for reducing postoperative corneal astigmatism. In the days after cataract surgery, anterior segment biometry may provide hints for the detection of conditions such as intraocular pressure (IOP) spikes, whose prevalence is associated with the dimensions of the anterior segment [7].

Accurate anterior segment biometry before cataract surgery is especially emphasized for patients with high axial myopia [1]. Characterized by excessive ocular elongation, high axial myopia is a vision-threatening ocular disorder defined as an axial length (AL) longer than 26.5 mm [8]. Though the postoperative refractive accuracy of cataract surgery in high myopes is challenging, [1] considering more anterior segment parameters has enabled fourth- and fifth-generation formulas to yield better refractive outcomes than formulas from prior generations [2, 3]. Furthermore, the anterior chamber is often unstable in high myopic eyes, with a large, floppy capsular bag and occasional zonular weakness. These structural characteristics not only make highly myopic eyes surgically challenging, but also necessitate choosing an IOL of high in-the-bag stability [9]. In addition, high axial myopia is associated with compromised biomechanical properties of the cornea [10]. The weak and deformable corneas may bring difficulty in creating a watertight seal at the site of the corneal incision, leading to postoperative leakage of aqueous humor. To compensate compromised corneal biomechanical properties, corneal biometric characteristics should be taken into consideration when implementing cataract surgeries in high myopes, particularly for the siting, making, and sealing of corneal cataract incisions.

However, despite the great significance of anterior segment biometry for refractive cataract surgery, there have been few studies on the relationship between high axial myopia and anterior segment biometric characteristics [1, 11, 12]. Some previous studies on healthy subjects have partially explored the link between myopic refraction and anterior segment biometry [13–16]. However, they were mostly implemented in Western and South Asian countries and showed considerable inconsistencies amongst themselves. Q value, for instance, several studies have shown its positive correlation with the degree of myopia, [13] while other studies have reported more negative Q values in subjects with a higher degree of myopia [14]. As for other anterior segment biometric parameters, Dong J et al. reported deep ACD, lower CRP, and greater CA in the high myopia group than in the normal group [16]. Yet, another large, cross-sectional, population-based study of Chinese individuals reported shallow ACD in persons with myopia, even those with long ALs [15]. Furthermore, few studies have investigated the characteristics of corneal astigmatism in high myopic eyes using adult samples.

We hypothesized that high axial myopia is linked to certain alterations in anterior segment biometry. In this study, using a large sample size consisting of 3438 adult Chinese cataract patients, we aimed to investigate the association of high axial myopia and various anterior segment biometric parameters, in addition to revealing the influence of ocular elongation on anterior segment biometric parameters, thereby improving the surgical practice of refractive cataract procedures and possibly informing the selection of IOLs and the calculation of their power in people with high myopia.

## Methods

### Study design and objective

This was a cross-sectional, hospital-based study carried out at the Eye & ENT Hospital of Fudan University (Shanghai, China) with continuous enrollment after October 2015. The research protocols were approved by the Institute Ethics Committee of the Eye & ENT Hospital of Fudan University (Shanghai, China) and conformed to the tenets of the Declaration of Helsinki. Informed consent was obtained from each subject.

### Patients

The subjects in this retrospective study were consecutive patients who were scheduled for cataract surgery at the Eye & ENT Hospital of Fudan University (Shanghai, China) from 2016 to 2020. The Eye & ENT Hospital of Fudan University is the largest tertiary specialty hospital with an ophthalmic clinic in Shanghai; this institution has the highest patient output and academic productivity among all eye clinics in Shanghai and its surrounding regions. For statistical analysis, high axial myopia was defined as an AL equal to or greater than 26.50 mm [8]. All the eyes in this study were studied in pre-surgical stage.

All the subjects agreed to preoperative examinations. Only adults of Han ethnicity were enrolled to avoid potential bias attributable to ethnic factors. The patients with the lens-related systemic syndromes, such as Marfan, Ehrler-Danlos, Weil-Marchesani, and homocystinuria, were not included in this study. Only one randomly selected eye of each subject was included for the analysis. The randomization was achieved by the generation of random number (OD, 0; OS, 1) in Microsoft Excel (Microsoft Corporation, Redmond, Washington). An additional inclusion criterion for this study was the availability and quality of anterior segment biometry records. Subjects who had low- quality Oculus Pentacam HR and Zeiss IOLMaster 500 scans were excluded. A total of 4305 subjects met the inclusion criteria and were originally enrolled, including 3354 subjects with high axial myopia (AL, ≥26.50 mm) and 951 subjects without (AL, < 26.50 mm).

The exclusion criteria of this study were previous ocular surgeries or trauma, corneal diseases, glaucoma, lens dislocations, and other ocular disorders that may have affected the anterior eye. Besides, the patients with pathological myopia featuring severe maculopathy were also excluded because of their different characteristics of postoperative refractive success [17–19]. After taking the exclusion criteria into consideration, 867 eyes got excluded from 4305 eyes. Finally, 3438 eyes were used for the statistical analysis.

### Ocular and systemic examinations

Each subject in our study underwent detailed preoperative examinations, including the determination of uncorrected and best corrected visual acuity, noncontact tonometry (TonoRef II, Nidek), a slit-lamp examination before and after pupil dilation, B ophthalmic ultrasonography (Medical Aviso, Quantel), rotating Scheimpflug photography (Pentacam HR, Oculus), optical interferometry (IOLMaster 500, Zeiss), and fundus optical coherence tomography (Cirrus HD-OCT 5000, Zeiss). The examinations were implemented by highly experienced technicians blinded to the patient assignments.

The AL was evaluated using a Zeiss IOLMaster 500. The IOP was measured with a Nidek TonoRef II. The other characteristics of the anterior segment, including the corneal topography and ACD, were assessed with an Oculus Pentacam HR. Total corneal refractive power (TCRP) was determined on two perpendicular axes (the flat meridian and the steep meridian), and a mean value was calculated by averaging these measurements. The Q value was calculated at the central 6-mm-diameter ring with reference to the corneal apex. The measurements of nasal (NCT), temporal (TCT), inferior (ICT), and superior corneal thickness (SCT) were recorded at the central 8-mm-diameter ring according to polar coordinates. The automatic mode of measurements was used in all devices to acquire the variables. The operators only needed to adjust the devices to the subject’s eye and start the measurement. When the instrument was correctly aligned, the measurement would automatically begin. For the evaluation of IOP, an average of three readings from Nidek TonoRef II was recorded for each patient.

As part of routine preoperative inspections, systemic examinations, including electrocardiography, chest X-ray, routine blood tests, and blood biochemistry, were performed in each subject. Each subject’s heart rate and arterial blood pressure were also measured. The diagnoses of arterial hypertension and diabetes mellitus were made by analyzing past history and preoperative testing.

### Statistical analysis

First, the distributions of ocular biometric parameters are described as the means ± standard deviations (SD) according to age and sex (Table 1). The effects of age and sex (female, 1; male, 0) on ocular biometric parameters and the relationships between the biometric parameters were assessed with Pearson’s correlation analysis. Pearson’s correlation coefficients (r) were calculated to evaluate the relationships.

**Table 1:** Distribution of ocular biometric parameters in the study population

Age Group (years)	Q Value (*n* = 2872)	TCRP (D) (*n* = 3057)	CA (D) (*n* = 3057)	WTW (mm) (*n* = 2461)	CCT (μm) (*n* = 2878)	ACD (mm) (*n* = 2935)	IOP (mmHg) (*n* = 2989)	AL (mm) (*n* = 3438)
Female								
≤ 59 (*n* = 670)	− 0.24 ± 0.16	43.4 ± 1.3	1.12 ± 0.71	11.40 ± 0.40	536.4 ± 30.5	2.90 ± 0.46	15.6 ± 3.1	29.21 ± 2.82
60–69 (*n* = 840)	− 0.18 ± 0.18	43.7 ± 1.2	1.09 ± 0.70	11.32 ± 0.41	537.3 ± 32.5	2.87 ± 0.55	15.1 ± 3.1	28.37 ± 2.75
≥ 70 (*n* = 428)	− 0.16 ± 0.21	43.7 ± 1.2	1.14 ± 0.75	11.30 ± 0.37	534.7 ± 31.5	2.79 ± 0.50	14.4 ± 3.3	27.67 ± 2.48
All (*n* = 1938)	− 0.19 ± 0.18	43.6 ± 1.2	1.11 ± 0.71	11.34 ± 0.40	536.4 ± 31.6	2.86 ± 0.51	15.1 ± 3.2	28.50 ± 2.78
Male								
≤ 59 (*n* = 554)	− 0.21 ± 0.13	42.9 ± 1.2	1.07 ± 0.69	11.56 ± 0.42	546.2 ± 32.7	3.05 ± 0.48	16.1 ± 3.7	29.06 ± 2.71
60–69 (*n* = 558)	− 0.17 ± 0.18	43.2 ± 1.2	0.97 ± 0.61	11.48 ± 0.43	547.4 ± 33.7	2.93 ± 0.43	15.4 ± 3.4	28.68 ± 2.70
≥ 70 (*n* = 388)	− 0.16 ± 0.21	43.3 ± 1.2	1.01 ± 0.66	11.50 ± 0.44	545.9 ± 32.2	2.91 ± 0.49	14.5 ± 3.2	27.80 ± 2.46
All (*n* = 1500)	− 0.18 ± 0.17	43.1 ± 1.2	1.02 ± 0.66	11.52 ± 0.43	546.6 ± 32.9	2.97 ± 0.47	15.4 ± 3.5	28.59 ± 2.69
Female and Male								
≤ 59 (*n* = 1224)	−0.23 ± 0.15	43.2 ± 1.3	1.10 ± 0.70	11.47 ± 0.41	540.8 ± 31.9	2.97 ± 0.48	15.9 ± 3.4	29.14 ± 2.77
60–69 (*n* = 1398)	− 0.18 ± 0.18	43.5 ± 1.2	1.04 ± 0.67	11.38 ± 0.43	541.3 ± 33.3	2.89 ± 0.51	15.2 ± 3.3	28.49 ± 2.74
≥ 70 (*n* = 816)	−0.16 ± 0.21	43.5 ± 1.2	1.07 ± 0.71	11.40 ± 0.41	539.9 ± 32.3	2.85 ± 0.50	14.4 ± 3.3	27.73 ± 2.47
All (*n* = 3438)	− 0.19 ± 0.18	43.4 ± 1.3	1.07 ± 0.69	11.42 ± 0.42	540.8 ± 32.6	2.91 ± 0.50	15.3 ± 3.3	28.54 ± 2.74
*r* (age)	0.17 ***	0.14 ***	−0.02	−0.10 ***	− 0.01	−0.10 ***	− 0.17 ***	−0.19 ***
*r* (sex)	−0.03	0.18 ***	0.07 ***	−0.20 ***	−0.15 ***	− 0.11 ***	−0.04 *	− 0.02

*Abbreviations*: A*CD* Anterior chamber depth, *AL* Axial length, *CA* Corneal astigmatism, *CCT* Central corneal thickness, D Diopter, *IOP* Intraocular pressure, *Q* Corneal asphericity coefficient, *r* Pearson’s correlation coefficient, *TCRP* Total corneal refractive power, *WTW* White-to-white corneal diameter

*P < 0.05, **P < 0.01, and ***P < 0.001 (two-tailed)

Second, the subjects were separated into three subgroups according to AL as follows: the subgroup with normal axial dimension (AL, ≥21.50 to < 24.50 mm), the subgroup with medium-long axial dimension (AL, ≥24.50 to < 26.50 mm), and the subgroup with long axial dimension (AL, ≥26.50 mm) [20]. Corneal astigmatism ≥1.5 D was considered an indication for the implantation of a toric intraocular lens during cataract surgery [21]. The cutoff value for intraocular pressure was set at 21 mmHg to distinguish ocular hypertension [22]. The characteristics of the patients in three subgroups were compared using one-way analysis of variance for quantitative variables and Pearson’s χ^2^ test for qualitative variables (Table 2). Furthermore, the normal and medium-long axial dimension subgroups in Table 2 were merged into the non-high axial myopia subgroup in Table 3. Intergroup significance between the non-high axial myopia subgroup (AL, < 26.50 mm) and high axial myopia subgroup (AL, ≥26.50 mm) was analyzed using Student’s t test and Pearson’s χ2 test (Table 3).

**Table 2:** Characteristics of subjects in axial length subgroups (One-Way Analysis of Variance and Pearson’s χ^2^ Test)

Characteristics	AL (Group) ^a^			*P* Value^e^
	Normal (*n* = 238)	Medium-long (*n* = 535)	Long (*n* = 2665)	
Age (years)	238 (66.2 ± 9.8) ^b^	535 (63.9 ± 11.3) **	2665 (61.5 ± 10.4) ***	< 0.001
AL (mm)	238 (23.37 ± 0.64)	535 (25.90 ± 0.51) ***	2665 (29.54 ± 2.19) ***	< 0.001
Q Value	195 (−0.14 ± 0.21)	451 (− 0.17 ± 0.16) *	2226 (− 0.20 ± 0.18) ***	< 0.001
TCRP (D)				
Flat Meridian	203 (43.35 ± 1.19)	476 (42.92 ± 1.27) ***	2378 (42.77 ± 1.25) ***	< 0.001
Steep Meridian	203 (44.33 ± 1.18)	476 (43.91 ± 1.34) ***	2378 (43.87 ± 1.34) ***	< 0.001
Mean Value	203 (43.87 ± 1.14)	476 (43.44 ± 1.26) ***	2378 (43.34 ± 1.25) ***	< 0.001
CA (D)	203 (0.98 ± 0.65)	476 (1.00 ± 0.67)	2378 (1.09 ± 0.70) *	0.005
Degree of CA				
< 1.5 D	163 (80.3)	378 (79.4)	1781 (74.9)	0.04
≥ 1.5 D ^c^	40 (19.7)	98 (20.6)	597 (25.1)	
Axis of CA				0.18
WTR	73 (36.0)	203 (42.7)	1028 (43.3)	
ATR	93 (45.8)	177 (37.3)	886 (37.3)	
OB	37 (18.2)	95 (20.0)	462 (19.4)	
WTW (mm)	150 (11.25 ± 0.36)	379 (11.43 ± 0.40) ***	1932 (11.43 ± 0.43) ***	< 0.001
CT (μm)				
Central	197 (532.4 ± 30.6)	453 (541.1 ± 31.4) **	2228 (541.5 ± 32.9) ***	0.001
Temporal	196 (645.9 ± 38.5)	453 (661.4 ± 42.4) ***	2219 (669.7 ± 46.3) ***	< 0.001
Nasal	182 (696.0 ± 45.6)	426 (697.1 ± 44.2)	2152 (688.5 ± 46.8) *	0.001
ACD (mm)	197 (2.52 ± 0.42)	460 (2.80 ± 0.46) ***	2278 (2.96 ± 0.49) ***	< 0.001
IOP (mmHg)	217 (14.7 ± 3.0)	449 (14.8 ± 3.2)	2323 (15.4 ± 3.4) **	< 0.001
Scale of IOP				0.002
< 21 mmHg	214 (98.6)	437 (97.3)	2196 (94.5) **	
≥ 21 mmHg ^d^	3 (1.4)	12 (2.7)	127 (5.5) **	

*Abbreviations*: *ACD* Anterior chamber depth, *AL* Axial length, *ATR* against-the-rule astigmatism, *CA* Corneal astigmatism, *CT* Corneal thickness, *D* Diopter, *IOP* Intraocular pressure, *OB* Oblique astigmatism, *Q* corneal asphericity coefficient, *TCRP* Total corneal refractive power, *WTR* With-the-rule astigmatism, *WTW* White-to-white corneal diameter

*P < 0.05, **P < 0.01, and ***P < 0.001 (two-tailed). The *P* values were from post hoc comparisons with the normal subgroup

^a^The division of AL subgroups: normal, 21.50 ≤ AL < 24.50 mm; medium-long, 24.50 ≤ AL < 26.50 mm; long, AL ≥26.50 mm

^b^For quantitative variables, the values are expressed as the number of eyes (mean ± standard deviation); for qualitative variables, the values are expressed as the number of eyes (proportion)

^c^Indication for toric intraocular lens implantation during cataract surgery

^d^Ocular hypertension

^e^The *P* values were from one-way analysis of variance and Pearson’s χ2 test

**Table 3:** Characteristics of Subjects with and without High Axial Myopia (Student’s *t* Test and Pearson’s χ2 Test)

Characteristics	AL (Group) ^a^		*P* Value
	Non-Highly Axial Myopia (*n* = 773)	Highly Axial Myopia (*n* = 2665)	
Age (years)	773 (64.6 ± 10.9) ^b^	2665 (61.5 ± 10.4)	< 0.001
AL (mm)	773 (25.12 ± 1.29)	2665 (29.54 ± 2.19)	< 0.001
Q Value	646 (−0.16 ± 0.18)	2226 (− 0.20 ± 0.18)	< 0.001
TCRP (D)			
Flat Meridian	679 (43.05 ± 1.26)	2378 (42.77 ± 1.25)	< 0.001
Steep Meridian	679 (44.04 ± 1.31)	2378 (43.87 ± 1.34)	0.003
Mean Value	679 (43.56 ± 1.24)	2378 (43.34 ± 1.25)	< 0.001
CA (D)	679 (0.99 ± 0.66)	2378 (1.09 ± 0.70)	0.001
Degree of CA			
< 1.5 D	541 (79.7)	1781 (74.9)	0.01
≥ 1.5 D ^c^	138 (20.3)	597 (25.1)	
Axis of CA			0.43
WTR	276 (40.7)	1028 (43.3)	
ATR	270 (39.8)	886 (37.3)	
OB	132 (19.5)	462 (19.4)	
WTW (mm)	529 (11.38 ± 0.39)	1932 (11.43 ± 0.43)	0.03
CT (μm)			
Central	650 (538.4 ± 31.4)	2228 (541.5 ± 32.9)	0.04
Temporal	649 (656.7 ± 41.9)	2219 (669.7 ± 46.3)	< 0.001
Nasal	608 (696.8 ± 44.6)	2152 (688.5 ± 46.8)	< 0.001
ACD (mm)	657 (2.72 ± 0.47)	2278 (2.96 ± 0.49)	< 0.001
IOP (mmHg)	666 (14.8 ± 3.1)	2323 (15.4 ± 3.4)	< 0.001
Scale of IOP			0.001
< 21 mmHg	651 (97.7)	2196 (94.5)	
≥ 21 mmHg ^d^	15 (2.3)	127 (5.5)	

*Abbreviations*: *ACD* Anterior chamber depth, *AL* Axial length, *ATR* Against-the-rule astigmatism, *CA* Corneal astigmatism, *CT* Corneal thickness, *D* Diopter, *IOP* Intraocular pressure, *OB* Oblique astigmatism, *Q* Corneal asphericity coefficient, *TCRP* Total corneal refractive power, *WTR* With-the-rule astigmatism, *WTW* White-to-white corneal diameter

^a^The division of AL subgroups: non-highly axial myopia, 21.50 ≤ AL < 26.5 mm; highly axial myopia, AL ≥26.50 mm

^b^For quantitative variables, the values are expressed as the number of eyes (mean ± standard deviation); for qualitative variables, the values are expressed as the number of eyes (proportion)

^c^Indication for toric intraocular lens implantation during cataract surgery

^d^Ocular hypertension

Third, the associations between high axial myopia and the biometric parameters of the anterior segment were assessed using a multivariate-adjusted logistic model (Table 4) [23]. After adjustment for sex, age, diabetes mellitus, and arterial hypertension, [23, 24] the odds ratios (ORs) and their 95% confidence intervals (CIs) were calculated both continuously and after categorizing the values into quartiles. The *P* values for trend associations were also obtained.

**Table 4:** Association of High Axial Myopia with Anterior Segment Biometry (Multivariate-Adjusted Logistic Model)

Biometric Parameter	High Axial Myopia	
	*OR* (95% *CI*) ^a^	*P* Value
Q Value		
Quartile 1	1.0 (Ref)	–
Quartile 2	0.91 (0.66, 1.25)	0.55
Quartile 3	0.97 (0.71, 1.33)	0.87
Quartile 4	0.64 (0.47, 0.88)	0.005
	*P* = 0.009 (Trend)	
Per 1-unit increase	0.42 (0.23, 0.79)	0.007
TCRP (D)		
Quartile 1	1.0 (Ref)	–
Quartile 2	1.10 (0.81, 1.50)	0.55
Quartile 3	0.72 (0.54, 0.97)	0.03
Quartile 4	0.72 (0.53, 0.96)	0.03
	*P* = 0.003 (Trend)	
Per 1-D increase	0.86 (0.79, 0.94)	0.001
CA (D)		
Quartile 1	1.0 (Ref)	–
Quartile 2	1.04 (0.78, 1.39)	0.80
Quartile 3	1.21 (0.92, 1.59)	0.18
Quartile 4	1.32 (0.99, 1.75)	0.06
	*P* = 0.04 (Trend)	
Per 1-D increase	1.20 (1.03, 1.41)	0.02
WTW (mm)		
Quartile 1	1.0 (Ref)	–
Quartile 2	0.81 (0.59, 1.11)	0.19
Quartile 3	1.05 (0.76, 1.45)	0.76
Quartile 4	1.43 (0.99, 2.08)	0.06
	*P* = 0.04 (Trend)	
Per 1-mm increase	1.38 (1.03, 1.85)	0.03
ACD (mm)		
Quartile 1	1.0 (Ref)	–
Quartile 2	2.16 (1.63, 2.86)	< 0.001
Quartile 3	2.63 (1.96, 3.51)	< 0.001
Quartile 4	4.68 (3.35, 6.54)	< 0.001
	*P* < 0.001 (Trend)	
Per 1-mm increase	3.25 (2.51, 4.22)	< 0.001
IOP (mmHg)		
Quartile 1	1.0 (Ref)	–
Quartile 2	1.20 (0.91, 1.60)	0.20
Quartile 3	1.26 (0.95, 1.68)	0.11
Quartile 4	1.40 (1.04, 1.88)	0.03
	*P* = 0.03 (Trend)	
Per 1 mmHg increase	1.06 (1.02, 1.09)	0.001

*Abbreviations*: *ACD* Anterior chamber depth, *CA* Corneal astigmatism, *CI* Confidence interval, *D* Diopter, *IOP* Intraocular pressure, *OR* Odds ratio, *Q* Corneal asphericity coefficient, *TCRP* Total corneal refractive power, *WTW* White-to-white corneal diameter

^a^Adjusted for age, sex, arterial hypertension, and diabetes mellitus

Fourth, we used a multivariate-adjusted linear model to evaluate the correlations between AL and the biometric parameters of the anterior segment. After adjusting for age, sex, diabetes mellitus, and arterial hypertension, the standardized linear regression coefficients (β), linear regression coefficients (B) and 95% CIs were calculated using the model.

In this study, the *P* values were calculated with two-sided tests, and the significance threshold was set at 5% in all statistical analyses. IBM SPSS Statistics (version 23.0) was used for statistics and graphics.

## Results

### Study population

A total of 3438 eyes were analysed. The mean age of the subjects was 62.2 ± 10.6 years (range, 18–94 years; median, 63.0 years), and 56.4% were female. There were 2665 patients (77.5%) with high axial myopia (AL, ≥26.5 mm) and 773 patients without (AL, < 26.5 mm). The mean AL of the study population was 28.54 ± 2.74 mm (range, 21.58–37.11 mm; median, 28.25 mm). Table 1 presents the Q value, TCRP, TCA, WTW, central CT (CCT), ACD, IOP, and AL data for the subjects (mean ± SD) according to age and sex.

### Correlation of ocular biometry

A Pearson’s correlation analysis was made to investigate the basic associations between the variables and to give hints for further multivariate-adjusted analysis (Table 1). Younger age was correlated with a more negative Q value, greater TCRP, greater WTW, deeper ACD, higher IOP, and greater AL. Women showed lower WTW, CCT, ACD, and IOP than men, whereas TCRP and TCA values were lower in men than in women.

Pearson’s correlation analysis also revealed the relationships between the anterior segment biometric parameters. A very low correlation was observed between a more negative Q value and a lesser WTW (*r*, 0.14; *P* <  0.001). Low correlations were detected between the CCT and IOP (*r*, 0.26; *P* <  0.001) and between the ACD and WTW (*r*, 0.25; *P* <  0.001). A moderate inverse correlation was detected between TCRP and WTW (*r*, − 0.41; *P* <  0.001).

Furthermore, the analysis for the correlations between the AL and anterior segment biometric parameters was made within subgroups categorized according to AL. In the high axial myopia subgroup (AL, ≥ 26.5 mm), the AL showed a statistically significant inverse relationship with Q value (*r*, − 0.11; *P* <  0.001) and NCT (*r*, − 0.08; *P* <  0.001), and a significant direct association with TCT (*r*, 0.12; *P* <  0.001) and IOP (*r*, 0.07; *P* <  0.001). In the non-high axial myopia subgroup (AL, < 26.5 mm), the effect of ocular elongation on Q value (*r*, − 0.08; *P* <  0.05) and TCT (*r*, 0.21; *P* <  0.001) remained significant. Besides, greater AL was correlated with lower TCRP (*r*, − 0.18; *P* <  0.001), thicker CCT (*r*, 0.15; *P* <  0.001), wider WTW (*r,* 0.21; *P* <  0.001), and deeper ACD (*r*, 0.32; *P* <  0.001).

### Comparison of anterior segment biometry between axial length subgroups

**Fig. 1:**
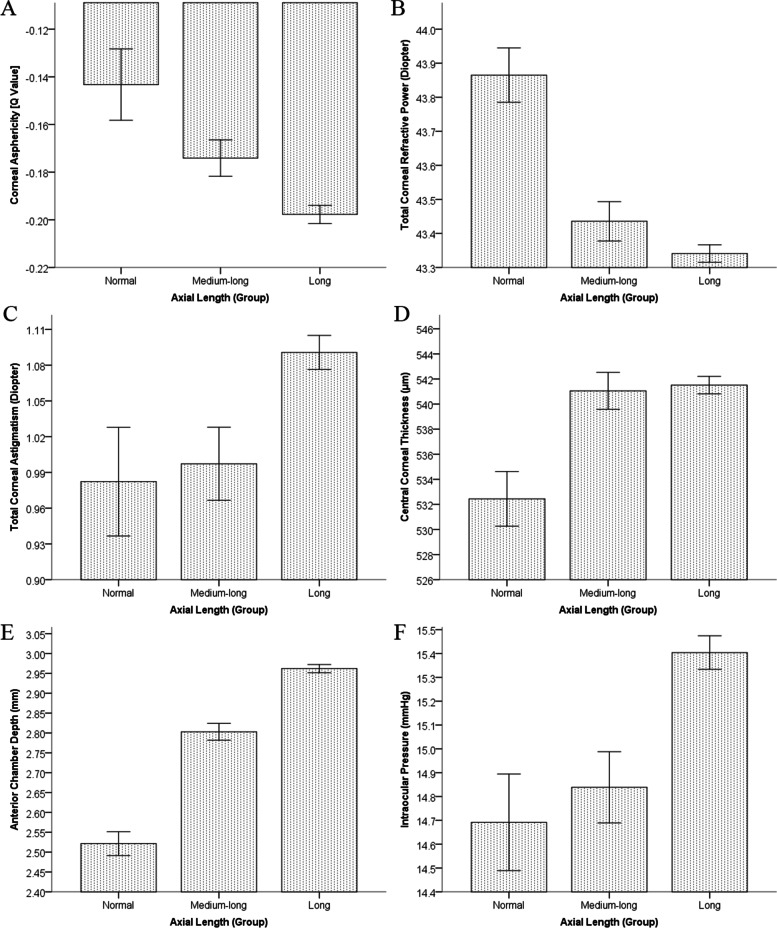
Histograms comparing anterior segment biometric parameters between axial length subgroups. **A**. Corneal asphericity [Q value]; **B**. Total corneal refractive power; **C**. Total corneal astigmatism; **D**. Central corneal thickness; **E**. Anterior chamber depth; **F**. Intraocular pressure. Error bars represent the standard error

Table 2 shows the results of one-way analysis of variance and Pearson’s χ^2^ test for the comparison of anterior segment biometry between three subgroups separated according to AL. The mean age of the subgroup with normal axial dimensions was significantly greater than that of the subgroups with medium-long and long axial dimensions. The subgroup of eyes with a long AL had more negative Q values, lower TCRP, greater TCA, thicker CCT and TCT, thinner NCT, wider WTW, deeper ACD, and higher IOP than the subgroup with a normal axial dimension (Fig. 1).

Table 2 also shows the Pearson’s χ^2^ test results for the qualitative parameters. The eyes for which toric intraocular lenses were indicated (TCA, ≥1.5 D) included a higher proportion of eyes with long ALs than of eyes with medium-long ALs. However, the proportions of eyes with with-the-rule astigmatism, against-the-rule astigmatism, and oblique astigmatism did not differ significantly between the three AL subgroups. The long axial dimension subgroup had a higher prevalence of ocular hypertension (IOP, ≥21 mmHg) than the normal axial dimension subgroup.

Table 3 shows the results of Student’s t test and Pearson’s χ^2^ test for the comparisons between non-high axial myopia subgroup (AL, < 26.5 mm) and high axial myopia subgroup (AL, ≥ 26.5 mm). According to the Student’s t test, the subgroup with high axial myopia had more negative Q values, lower TCRP, greater TCA, thicker CCT and TCT, thinner NCT, wider WTW, deeper ACD, and higher IOP than the subgroup of non-high axial myopia. According to the Pearson’s χ^2^ test, the eyes for which toric intraocular lenses were indicated (TCA, ≥1.5 D) included a higher proportion of eyes with high axial myopia than of eyes without. However, the proportions of eyes with with-the-rule astigmatism, against-the-rule astigmatism, and oblique astigmatism did not differ significantly between the two subgroups. The highly axial myopia subgroup had a higher prevalence of ocular hypertension (IOP, ≥21 mmHg) than the non-high axial myopia subgroup.

### Association between high axial myopia and anterior segment biometry

A multivariate-adjusted logistic regression model was used to investigate the associations between the presence of high axial myopia and anterior segment biometric parameters. Table 4 summarizes the relationship results of anterior segment biometric parameters with high axial myopia in a multivariate-adjusted logistic model. After adjusting for age, sex, diabetes mellitus, and arterial hypertension, the characteristics independently associated with high axial myopia included a more negative Q value (*OR*, 0.42; 95% *CI*, 0.23–0.79; *P* <  0.01 per unit increase), lower TCRP (OR, 0.86; 95% *CI*, 0.79–0.94; *P* <  0.01 per diopter increase), greater TCA (*OR*, 1.20; 95% *CI*, 1.03–1.41; *P* <  0.05 per diopter increase), wider WTW (*OR*, 1.38; 95% *CI*, 1.03–1.85; *P* <  0.05 per millimeter increase), deeper ACD (*OR*, 3.25; 95% *CI*, 2.51–4.22; *P* <  0.001 per millimeter increase), and higher IOP (*OR*, 1.06; 95% *CI*, 1.02–1.09; *P* <  0.01 per mmHg increase). After the values were categorized into quartiles, the associations remained significant in a trend analysis. No association was found between CCT and high axial myopia in the multivariate-adjusted logistic model (*OR*, 1.00; 95% *CI*, 0.99–1.01; *P* = 0.07 per micrometer increase).

### Association between axial length and anterior segment biometry

A multivariate-adjusted linear regression model was used to investigate the associations between AL and anterior segment biometric parameters. After adjustment for age, sex, arterial hypertension and diabetes mellitus, AL increased with significantly thicker TCT, thinner NCT, more negative Q value, lower TCRP, greater TCA, deeper ACD, and higher IOP.

For each millimeter increase in AL, the mean variation in TCT was + 2.3 μm (95% *CI*, 1.5–3.0 μm; *β*, 0.14; *P* <  0.001), in NCT was − 2.1 μm (95% *CI*, − 2.9 to − 1.4; *β*, − 0.13; *P* <  0.001), in the Q value was − 0.007 (95% *CI*, − 0.010 to − 0.004; *β*, − 0.11; *P* <  0.001), in flat TCRP was − 0.03 D (95% *CI*, − 0.05 to − 0.02; *β*, − 0.08; *P* < 0.01), and the mean TCRP was − 0.03 D (95% *CI*, − 0.04 to − 0.01; *β*, − 0.06; *P* < 0.05), in TCA was + 0.02 D (95% *CI*, 0.01–0.03; *β*, 0.08; *P* < 0.001), ACD was + 0.03 mm (95% *CI*, 0.02–0.04; *β*, 0.17; *P* < 0.001), and IOP was + 0.08 mmHg (95% *CI*, 0.03–0.13; *β*, 0.07; *P* < 0.01). No correlation was found between AL and steep TCRP in the multivariate-adjusted linear regression model (*B*, − 0.02; 95% *CI,* − 0.04 to + 0.01; *β*, − 0.03; *P* > 0.05).

## Discussion

This is the largest study (number of eyes = 3438) on the associations between high axial myopia and anterior segment biometry in cataractous eyes. Various parameters were extensively analyzed to offer a panorama of the anterior segment in highly myopic patients with cataracts. Here, we have shown the associations of high axial myopia with lower TCRP, more negative Q values, greater TCA, wider WTW, deeper ACD, and higher IOP. Thus, a comprehensive model featuring corneal flattening, increased TCA, anterior segment enlargement, and IOP elevation could be built for highly myopic eyes with cataracts. The findings may inform the choice of IOLs and the calculation of their power, help improve the surgical practice of refractive cataract procedures, and provide useful information on the centration and stability of IOLs.

In our study, longer ALs were observed in younger patients. The inverse relationship between age and AL may result from the early onset of cataracts in long eyes, which has been identified in previous large population-based studies [25, 26].

For IOL power calculation, the ACD, WTW, CRP, and Q value are four important parameters deserving special attention for IOL formula optimization in highly myopic eyes. Multivariate-adjusted logistic analysis showed that among the various parameters examined herein, ACD, WTW, CRP and Q value were four parameters associated with high axial myopia. Therefore, the characteristic anatomical deformities of long eyes may include a deep anterior chamber, wide corneal diameter, flat corneal apex, and great peripheral corneal flatness. Currently, the IOL formulas that take into account ACD, WTW, and CRP include several fourth-generation formulas, such as the Holladay II formula, and some fifth-generation formulas, such as the Barrett Universal II (BUII), Olsen, Hoffer H-5, Radial Basis Function (RBF) Calculator, and VRF formulas [27]. Compared with third-generation formulas using only AL and CRP, these modern formulas have led to remarkable improvements in refractive accuracy, especially in long eyes (AL > 26.0 mm) [2]. Even for eyes with an AL greater than 30.0 mm, the BUII and Olsen formulas still achieved good refractive outcomes [28]. To date, the Panacea formula is the only formula that enables cataract surgeons to enter the Q value [27]. In a study by Woong-Joo Whang on the optimization of IOL formulas, the accuracy of the Haigis-L formula was enhanced after taking into consideration the Q value at preoperative stage [29]. Therefore, we recommend creating a new IOL power formula in which Q value, WTW, ACD, and CRP are simultaneously considered.

No association was detected between CCT and high axial myopia in our study, which may explain why some fifth-generation IOL formulas that use CCT without utilizing WTW, such as the Emmetropia Verifying Optical (EVO) and Kane formulas, have not ranked among the most accurate formulas for people with high myopia [27].

The corneal pachymetric characteristics of highly myopic eyes may provide useful information for the selection of a corneal incision site. Our study reported thicker temporal cornea and thinner nasal cornea in longer eyes. Therefore, the temporal cornea may be the preferred site to make a watertight main incision in cataract surgery. For right-handed cataract surgeons making temporal main-port incisions, we recommend sitting on the superior or temporal side of the recumbent patient for right and left eye operations, respectively. Furthermore, a longer tunnel incision may be recommended for highly myopic eyes to facilitate watertight sealing and improve the impermeability of the wounds, especially when nasal incisions are made.

The increased WTW and ACD in the subjects with high axial myopia suggested anterior segment enlargement in highly myopic eyes. An enlarged anterior eye segment is often accompanied by a larger capsular bag size and a tendency toward dislocation and rotation of the IOL [5]. For premium IOLs such as toric, multifocal, and accommodative IOLs, good centration and stability are crucial for postoperative visual quality. Published papers reported better rotational stability of plate-haptic IOLs than loop-haptic IOLs in eyes with large capsular bags [6]. Therefore, for eyes with high myopia, plate-haptic IOLs may be a preferred option, especially when premium IOLs are used. In addition, if necessary, a capsular tension ring (CTR) can be used in highly myopic patients to assist IOL centration and reduce postoperative IOL rotation [30]. Anterior segment enlargement verified herein confirms the findings of several large cross-sectional studies, in which anterior chamber depth correlated positively with AL [13, 31]. In this study, the anterior chamber of highly myopic eyes (2.96 ± 0.50 mm) was deeper than reported in prior studies of Chinese subjects in Beijing (2.42 ± 0.34 mm) and Liwan (female, 2.39–2.4 mm; male, 2.56–2.62 mm) [32, 33]. The average WTW in eyes with high myopia (11.43 ± 0.45 mm) was greater than that of eyes with normative axial dimension in the Korean (11.33 ± 0.46 mm) and Chinese populations (11.36 ± 0.42 mm) [34, 35] and was comparable to a report on Chinese high myopia (11.45 ± 0.36 mm) [36].

For highly myopic eyes, the sclera is forcefully stretched and severely attenuated in highly myopic eyes due to excessive ocular elongation [37]. Similarly, the cornea in highly myopic eyes was shown to be weaker and more deformable with ocular elongation [10]. However, there has not yet been a fully satisfactory account of the mechanism causing the compromised biomechanical properties of the cornea. Our present study implies that, for eyes with high myopia, the forces acting upon the cornea from the enlarged anterior segment may be an underlying cause of decreased corneal rigidity. Interestingly, no correlation between WTW and AL was detected in the multivariate-adjusted linear regression model. This indicates that in eyes with high myopia, anterior segment enlargement is more prominent in the sagittal direction than in the transverse direction.

**Fig. 2:**
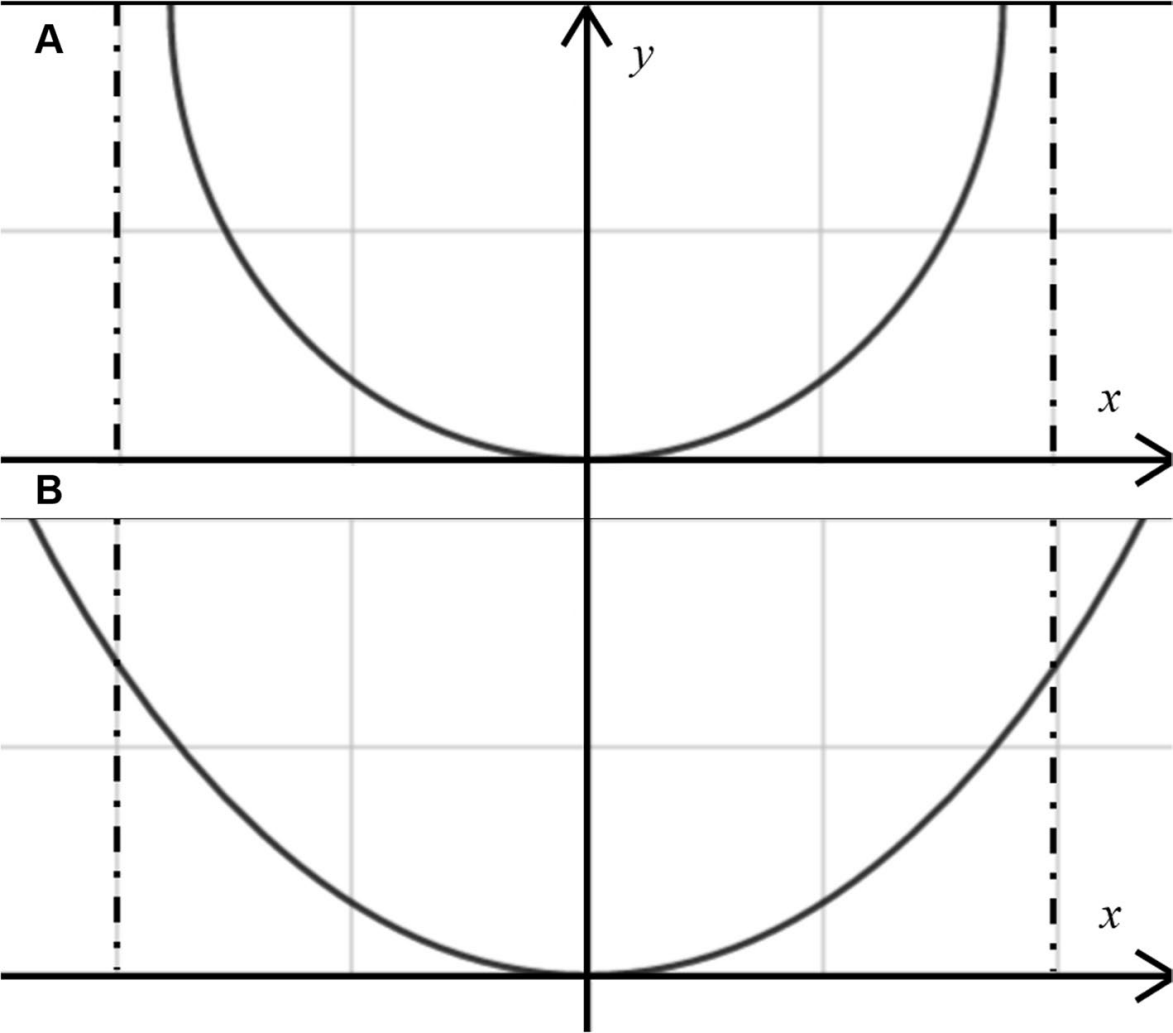
Schematic representation of the corneal optical contour of eyes without high axial myopia (**A**) and with high axial myopia (**B**). The corneas in highly myopic eyes had a more prolate optical contour with more rapid flattening toward the periphery than the corneas in eyes without high axial myopia

The anterior segment alterations in highly myopic eyes were also apparent as corneal flattening. The tendency for the cornea to have a more negative Q value and a flatter apex with globe elongation in this study confirms the results of previous research [14, 31]. Of conicoids with the same apical curvature, those with a more negative Q value will appear more prolate in optical contour, which indicates a greater degree of flattening toward the periphery. Therefore, in highly myopic eyes, the dome-shaped cornea is deformed to become less convex at the apex with greater flatness in the periphery (Fig. 2). Our findings also suggest that as AL increases, the cornea significantly flattens in the flat meridian rather than in the steep meridian, leading to an increased TCA. The eyes for which toric intraocular lenses were indicated (TCA, ≥1.5 D) included a higher proportion of eyes with high axial myopia (AL, ≥26.50 mm) than of eyes without (AL, < 26.50 mm). As a result, cataract patients with high axial myopia may be more likely to have an indication for toric IOL implantation during cataract surgery. Previous researches on the link between AL and TCA were mostly longitudinal studies on school-age children. In these studies, corneal astigmatism was correlated with the progression of ocular dimension and the development of ametropia [38, 39]. The association between long AL and great TCA in our study is in concordance with these previous reports. In addition, the proportions of eyes with with-the-rule astigmatism, against-the-rule astigmatism, and oblique astigmatism did not differ significantly among the different AL subgroups in our study (Table 2; Table 3).

Stretching and deformation of anterior chamber structures may also be an underlying cause of IOP elevation in highly myopic eyes. Our finding that intraocular pressure elevation is associated with high myopia is consistent with reports from several large, cross-sectional studies [22, 31], in which myopic eyes had an increased risk of ocular hypertension and multiple glaucoma subtypes. In the traditional view, more highly myopic eyes have a thinner trabecular meshwork and a greater diameter and area of Schlemm’s canal [40]. According to our study, a wider WTW and deeper ACD indicated an enlarged anterior segment in patients with high myopia. Generally, a stretched anterior chamber may influence mechanical support that the limbus provides to the trabecular meshwork and may result in changes in IOP. Further research is needed to identify the relationship between anterior segment enlargement and IOP elevation.

Our study had several limitations. First, its hospital-based cross-sectional design may have enhanced the degree of selection bias, and the results could not confirm a causal relationship. Therefore, our results need to be validated in a population-based study before they can be extrapolated to the entire population. Further prospective longitudinal follow-up studies are also required to confirm the causal relationship between high axial myopia and anterior segment alterations. Second, the Pearson’s correlations obtained in our study were not very strong. Those statistically significant while weak correlations did not imply that the associations were clinically significant as well. Therefore, more studies may be needed to establish the power of these correlations and build links of clinical significance. Third, the settings of the rotating Scheimpflug system were in manual mode with a reference sphere of 8-mm diameter. Therefore, the topographic parameters of the cornea were obtained from the central 8-mm diameter. Anterior segment optical coherence tomography may be helpful in collecting data from more peripheral locations. Fourth, the age distribution was different between different AL subgroups (Table 2; Table 3). This may enhance the degree of bias and influence the correlation analysis. We used the multivariate-adjusted models to adjust the effect of age and sex on anterior segment biometric parameters. However, it would be much nicer to balance the subgroups and avoid the bias by sample matching. Fifth, the IOP readings in this study were from non-contact tonometry (TonoRef II, Nidek) and were not adjusted by CCT. Despite high concordance between TonoRef II readings and Goldmann application tonometry, [41] the measurements of IOP may have been affected by CCT. Thus, further study in which the IOP readings are acquired with Goldmann application tonometry and are adjusted by CCT may be helpful to validate the associations of IOP in our study.

## Conclusions

In summary, in this large, hospital-based, cross-sectional study of 3438 subjects, high axial myopia was associated with corneal flattening (a flatter corneal apex and greater peripheral corneal flatness), increased TCA, anterior segment enlargement (a larger WTW and a deeper ACD) and IOP elevation. For high myopic eyes, we recommend creating a new IOL power formula in which the Q value, WTW, ACD, and CRP are simultaneously considered. Our findings may inform the choice of IOLs and the calculation of their power, help improve the surgical practice of refractive cataract procedures, and provide useful information on the centration and stability of IOLs.

## Data Availability

The datasets used and analysed during the current study are available from the corresponding author on reasonable request.

## Abbrevations

ACD: anterior chamber depth
AL: Axial length
B: Linear regression coefficients
BUII formula: Barrett Universal II formula
CA: Corneal astigmatism
CCT: Central corneal thickness
CI: Confidence interval
CRP: Corneal refractive power
CT: Corneal thickness
CTR: Capsular tension ring
EVO Formula: Emmetropia Verifying Optical formula
ICT: Inferior corneal thickness
IOL: Intraocular lens
IOP: Intraocular pressure
NCT: Nasal corneal thickness
OR: Odds ratio
Q value: Corneal asphericity coefficient
r: Pearson’s correlation coefficient
RBF Calculator: Radial Basis Function Calculator
SCT: Superior corneal thickness
SD: Standard deviation
TCA: Total corneal astigmatism
TCRP: Total corneal refractive power
TCT: Temporal corneal thickness
WTW: White-to-white corneal diameter
β: Standardized linear regression coefficient
